# Supplementary material for: An HLA-A*11:01-Binding Neoantigen from Mutated NPM1 as Target for TCR Gene Therapy in AML
Source: Cancers (Basel). 2021 Oct 27;13(21):5390. doi: 10.3390/cancers13215390 (PMC8582585; doi:10.3390/cancers13215390)
Supplement: Supplementary file 1 [file cancers-13-05390-s001.zip › cancers-1422109-supplementary.pdf]

cancers

MDPI

Supplementary Materials

An HLA-A*11:01-Binding Neoantigen from Mutated NPM1 as Target for TCR Gene Therapy in AML

Dyantha I. van der Lee, Georgia Koutsoumpli [1], Rogier M. Reijmers, Willy Honders, Rob C. M. de Jong, Dennis F. G. Remst, Tassilo L. A. Wachsmann, Renate S. Hagedoorn, Kees L. M. C. Franken, Michel G. D. Kester, Karl J. Harber, Lisanne M. Roelofsen, Annemiek M. Schouten, Arend Mulder, Jan W. Drijfhout, Hendrik Veelken, Peter A. van Veelen, Mirjam H. M. Heemskerk, Frederik Falkenburg and Marieke Griffioen

Table S1. Isolation of tetramer+ CD8 T-cells from healthy individuals.

Healthy Individual [1]	HLA-Allele	PBMC [2] ($\times 10^6$)	Sorted T-Cells [3]	Growing Clones
1	HLA-A11	575	224	122
2	HLA-A11	375	12	6
3	HLA-A11	4906	768	510
4	HLA-A11	340	369	139
5	HLA-A11	710	501	225
6	HLA-A3	520	156	82
7	HLA-A3	435	120	93
8	HLA-A3	605	91	67
9	HLA-A3	645	78	32
10	HLA-A3	440	62	30
11	HLA-A3	420	19	13
12	HLA-A3	225	60	37
13	HLA-A3	695	228	85
14	HLA-A3	475	136	80
15	HLA-A3	730	384	158
16	HLA-A3	450	113	35

[1] PBMCs from individuals 1-5 were stained with an HLA-A11 tetramer for the dNPM1 AVEEVSLRK peptide. For individuals 4 and 5, HLA-A11 tetramers for the dNPM1 CLAVEEVSLRK peptide and UV-exchange HLA-A11 tetramers for CLAVEEVSLRK and its cysteinylated variant C*LAVEEVSLRK were also added to the PBMCs. PBMCs from individuals 6-16 were stained with a mix containing HLA-A3 tetramers for AVEEVSLRK and CLAVEEVSLRK and UV-exchange HLA-A3 tetramers for CLAVEEVSLRK and cysteinylated C*LAVEEVSLRK. [2] Indicated is the amount of PBMCs from each healthy individual as used for T-cell isolation. [3] Indicated are numbers of tetramer+ CD8 T-cells that are sorted by flow cytometry from PBMCs.

Copyright: © 2021 by the authors. Licensee MDPI, Basel, Switzerland. This article is an open access article distributed under the terms and conditions of the Creative Commons Attribution (CC BY) license (http://creativecommons.org/licenses/by/4.0/).

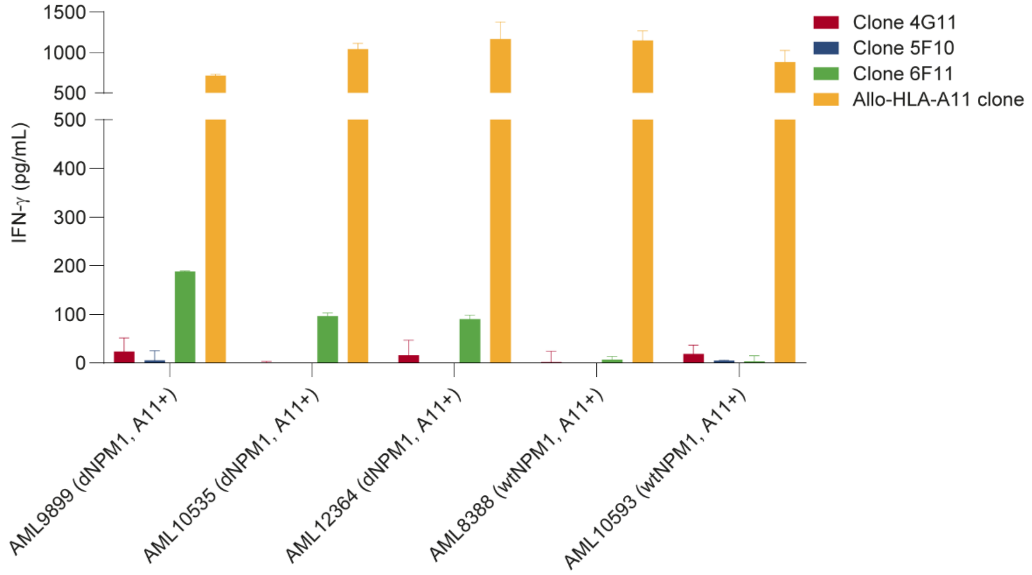

Figure S1. Clone 6F11 recognizes primary AMLs. T-cell clones 4G11 (red bars), 5F10 (blue bars) and 6F11 (green bars) were tested for recognition of 5 HLA-A11+ primary AMLs at an E:S ratio of 1:15. After overnight incubation, IFN-γ production was measured by ELISA. Clone 6F11 reacted against AMLs with dNPM1, while wtNPM1 AMLs were not recognized. Clones 4G11 and 5F10 did not show recognition of primary AMLs. An allo-HLA-A11 clone (orange bars) was included as positive control and reacted against all 5 HLA-A11+ AMLs. Bars represent mean + SD of duplicate wells.

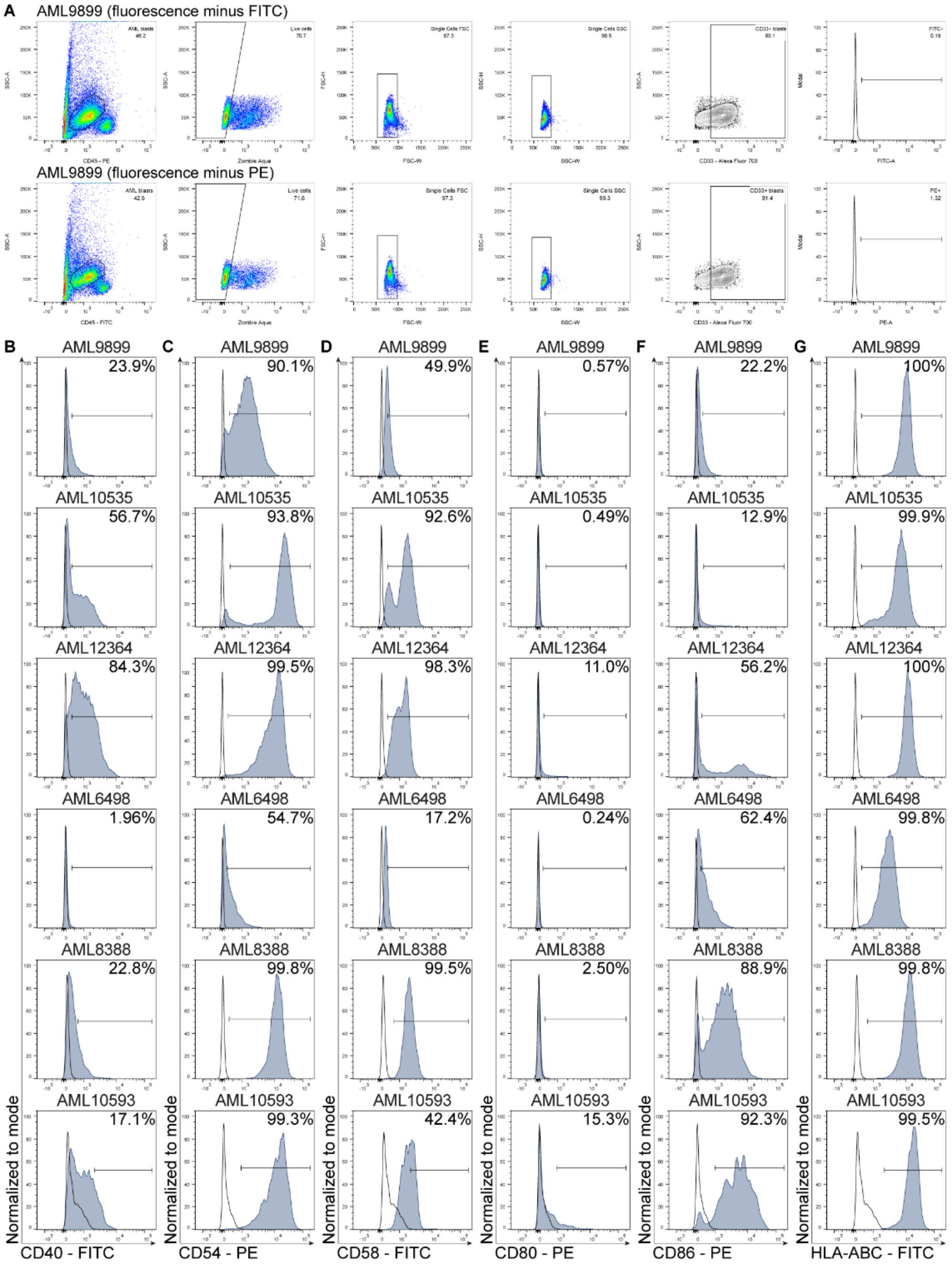

Figure S2. Surface expression of HLA class I, co-stimulation and adhesion molecules on primary AML. Primary AML samples were stained with a mix of anti-CD45 FITC or PE antibody, anti-CD33 Alexa Fluor 700 antibody and either anti-CD40 FITC, anti-CD54 PE, anti-CD58 FITC, anti-CD80 PE, anti-CD86 PE or anti-HLA class I FITC antibodies. Dead cells were excluded using Zombie Aqua. (**A**) Depicted is the gating strategy for measuring surface expression of HLA class I, co-stimulation and adhesion molecules on AML in a representative staining for AML9899. Using CD45, AML blasts ($CD45^{dim}$) were distinguished from other peripheral blood or bone marrow mononuclear cells ($CD45^{high}$). Next, CD33+ blasts were selected from viable single cells and expression of various adhesion and co-stimulation molecules within CD33+ AML blasts was determined using Fluorescence Minus One (FMO) controls without FITC (top panels) or PE (lower panels); (**B-G**) Staining for CD40 (**B**), CD54 (**C**) CD58 (**D**), CD80 (**E**), CD86 (**F**) and HLA-class I (**G**) on AML cells (blue histograms). Expression of CD40, CD54, CD58, CD80, CD86 and HLA class I was absent or low on dNPM1 AML6498, which correlated with absence of T-cell recognition. Black histograms depict FMO controls.

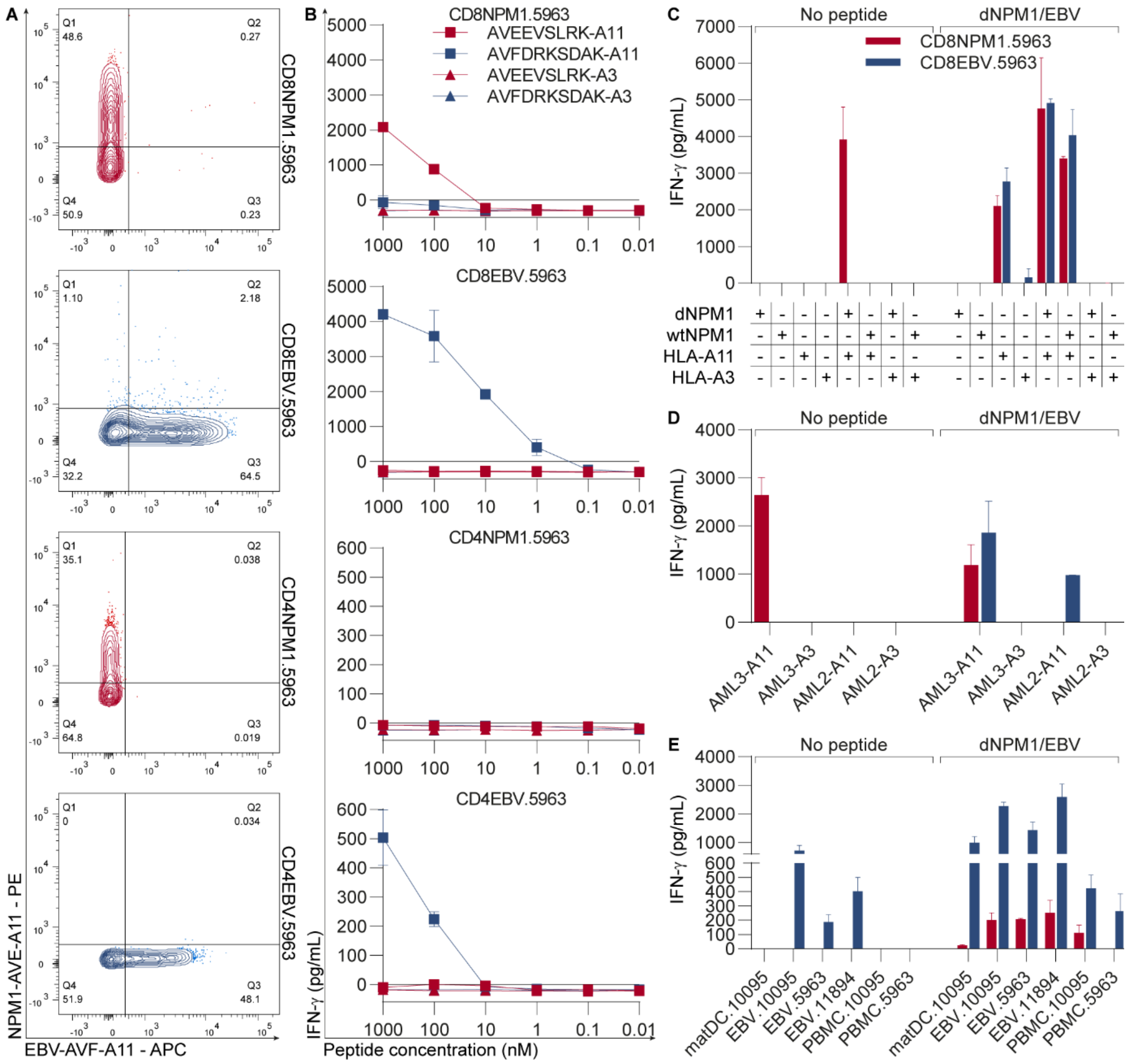

Figure S3. TCR gene transfer targeting dNPM1-derived AVEEVSLRK in HLA-A11. TCR-transduced T-cells were generated from donors 10095 and 5963 and tested as described in Figure 2. Results are shown for donor 5963. (**A**) Tetramer staining was assessed by flow cytometry. Staining with the NPM1-AVE-A11 tetramer was observed for 48.6% of CD8 and 35.1% of CD4 T-cells with the dNPM1 TCR (red) and 64.5% of CD8 and 48.1% of CD4 T-cells with the EBV TCR (blue) stained with the EBV-AVF-A11 tetramer; (**B**) T2 cells transduced with HLA-A11 (squares) or -A3 (triangles) were pulsed with AVEEVSLRK (red) or AVFDRKSDAK (blue) and incubated with T-cells. Recognition of AVEEVSLRK in HLA-A11 was seen for CD8 T-cells with the dNPM1 TCR, but not CD4 T-cells with the dNPM1 TCR. CD8 and CD4 T-cells with the EBV TCR recognized AVFDRKSDAK in HLA-A11; (**C**) K562 cells transduced with dNPM1 or wtNPM1 and HLA-A11 or -A3 were tested for recognition by CD8 T-cells. T-cells with the dNPM1 TCR (red bars) reacted against K562 cells with HLA-A11 and dNPM1. After peptide pulsing, K562 cells with HLA-A11 were recognized by T-cells with the dNPM1 TCR regardless of NPM1 transduction status, as well as by T-cells with the EBV TCR (blue bars); (**D**) CD8 T-cells were tested against OCI-AML3 with dNPM1 and OCI-AML2 with wtNPM1 transduced with HLA-A11 or -A3. T-cells with the dNPM1 TCR (red bars) secreted IFN-γ upon incubation with OCI-AML3 with HLA-A11, but not OCI-AML2. Peptide-pulsed OCI-AML2 with HLA-A11 was only recognized by T-cells with the EBV TCR (blue bars); (**E**) CD8 T-cells were incubated with autologous PBMCs, matDCs and EBV-LCLs from donors 10095 and 5963, and EBV-LCLs from donor 11894 from whom clone 6F11 was isolated. Healthy hematopoietic cells were not recognized by T-cells with the dNPM1 TCR (red bars), whereas T-cells with the EBV TCR (blue bars) reacted against EBV-LCLs. PBMCs and EBV-LCLs were also recognized by dNPM1 T-cells after peptide loading. Symbols and bars represent mean + SD of duplicate wells.

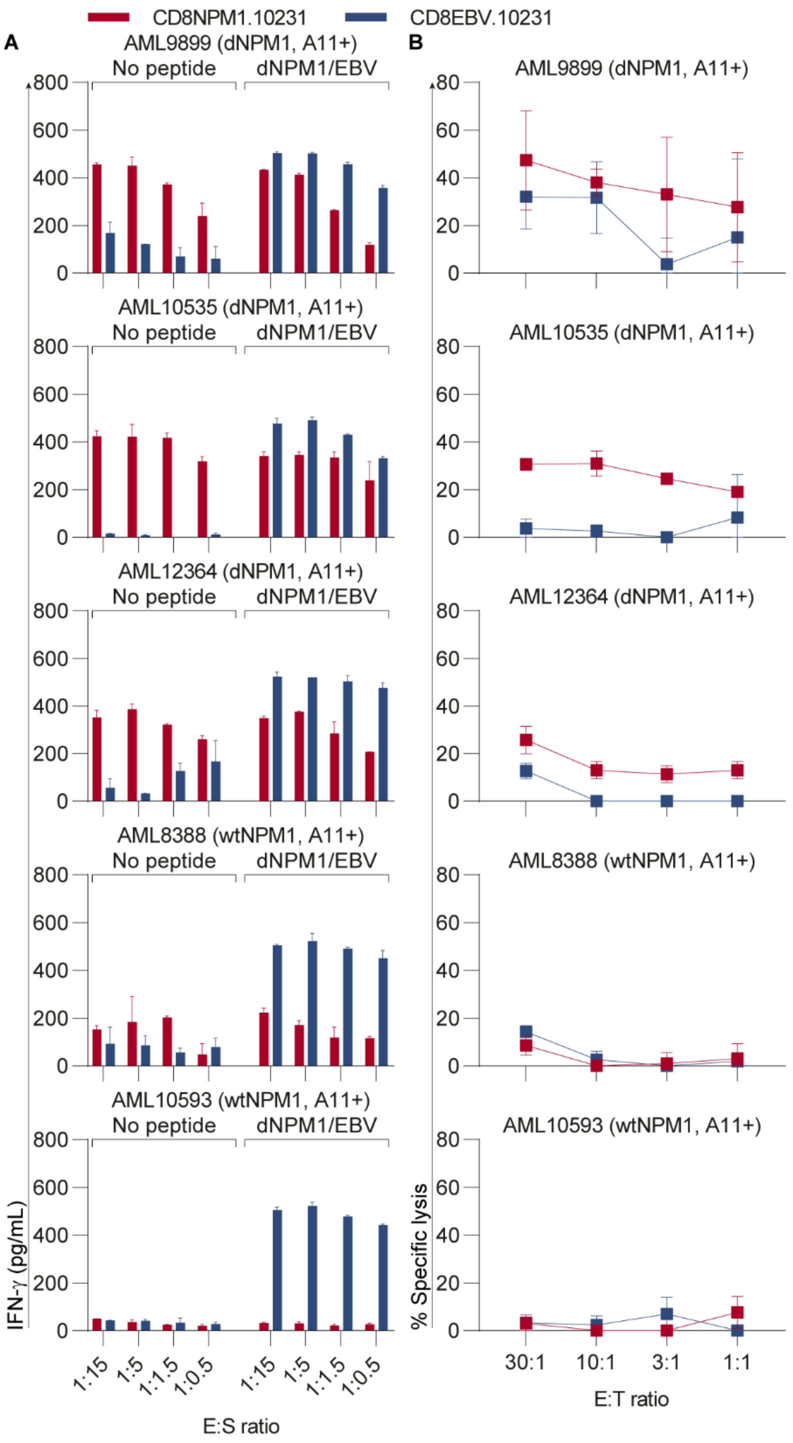

Figure S4. The HLA-A11-restricted dNPM1 TCR targets primary AML. CD8 T-cells transduced with the dNPM1 or EBV TCR were generated from donors 10095 and 10231 and tested as described in Figure 3. Results are shown for donor 10231. (**A**) T-cells were incubated with 5 HLA-A11+ primary AMLs. T-cells with the dNPM1 TCR (red bars) reacted against dNPM1 AMLs, while wtNPM1 AMLs were not recognized. T-cells with the EBV TCR (blue bars) were reactive against all 5 AMLs after peptide loading. Bars represent mean + SD of duplicate wells; (**B**) T-cell cytotoxicity against 5 HLA-A11+ primary AMLs was tested in a 9-h chromium-51 release assay. T-cells with the dNPM1 TCR (red line) lysed dNPM1 AMLs, while wtNPM1 AMLs were not killed. T-cells with the EBV TCR (blue line) did not show specific lysis of AMLs. Symbols represent mean ± SD of triplicate wells.

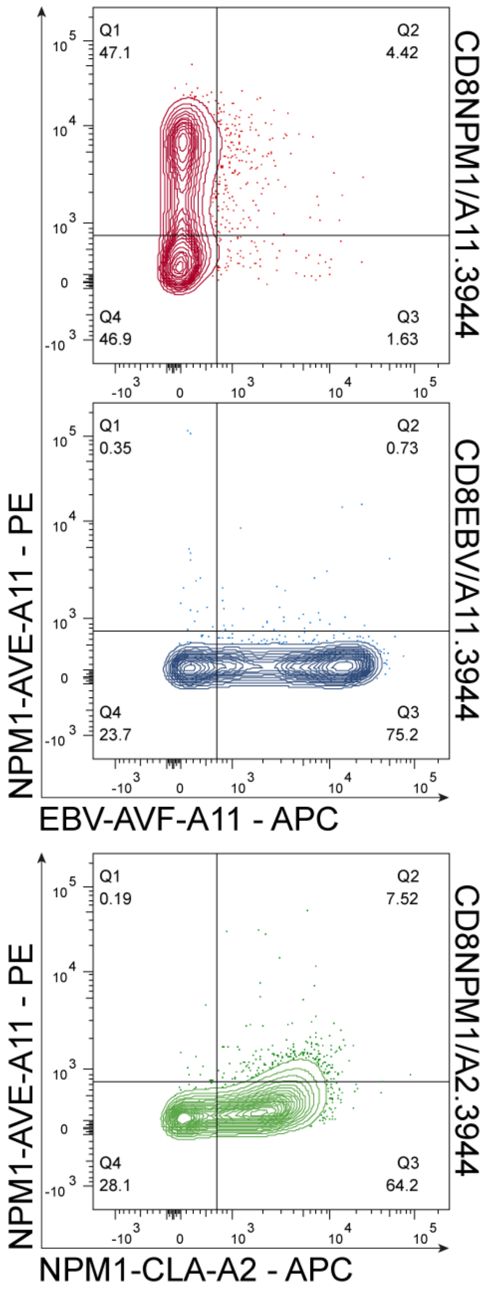

Figure S5. Tetramer staining of TCR-transduced T-cells as used for mouse experiments. CD8 T-cells from donor 3944 were transduced with the HLA-A11 dNPM1 or HLA-A11 EBV TCR or the HLA-A2 dNPM1 TCR. TCR-transduced T-cells were analyzed by FACS one day before injection into mice. T-cells with the HLA-A11 dNPM1 TCR (red), HLA-A11 EBV TCR (blue) and HLA-A2 dNPM1 TCR (green) stained with the NPM1-AVE-A11, EBV-AVF-A11 and NPM1-CLA-A2 tetramer, respectively.

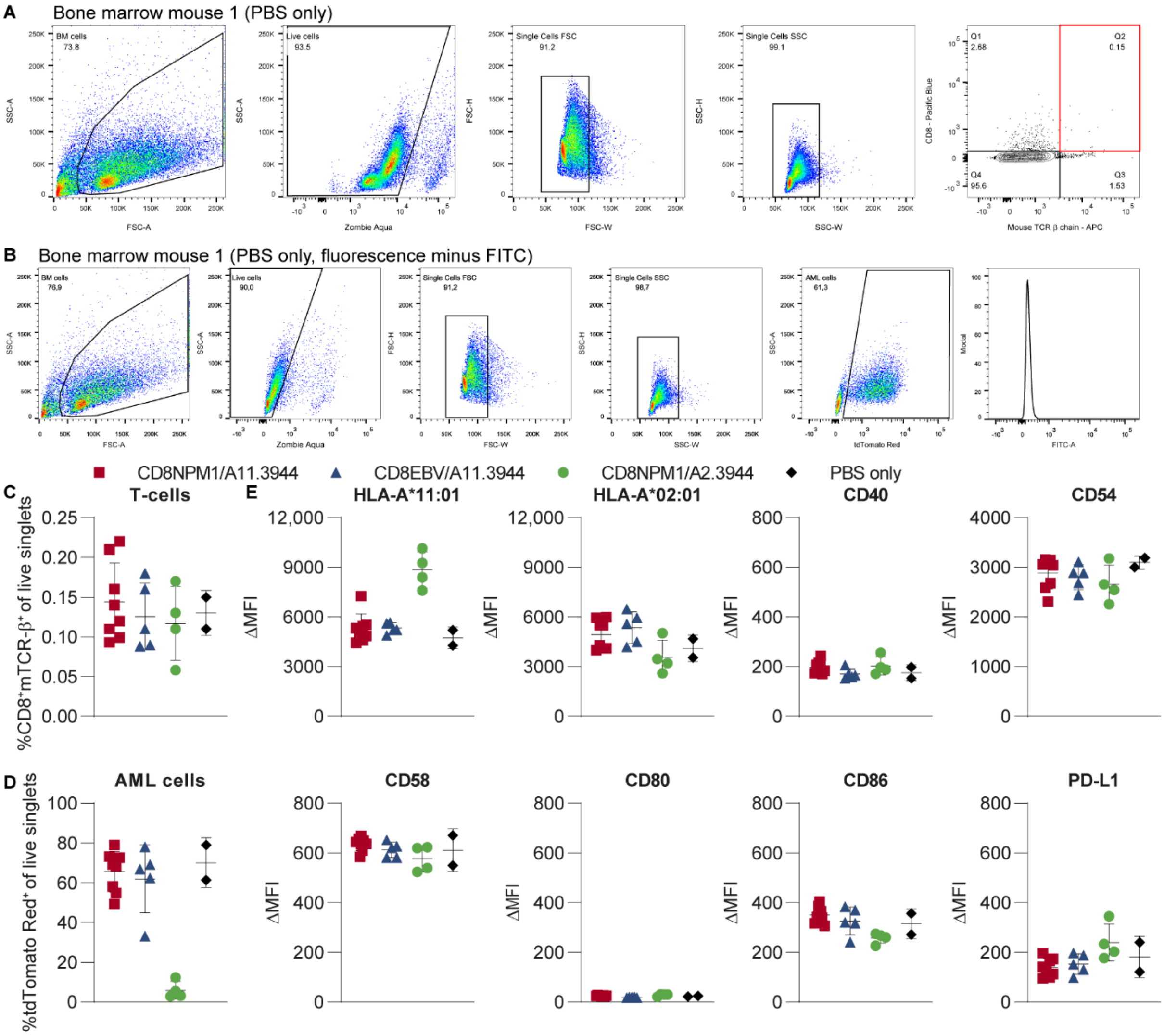

Figure S6. FACS analyses of mouse bone marrow samples. (**A**) Gating strategy for the analysis of T-cell persistence in bone marrow samples. Samples were stained with anti-human CD8 Pacific Blue and anti-mouse TCR β chain APC antibodies. Dead cells were excluded using Zombie Aqua. Depicted is a representative staining for bone marrow from mouse 1 treated with PBS. Within viable single cells, CD8+ mouse TCR β chain+ cells were selected (red rectangle); (**B**) Gating strategy to measure surface expression of HLA class I, co-stimulation and adhesion molecules on bone marrow samples. Samples were stained with anti-CD40 FITC, anti-CD54 APC, anti-CD58 FITC, anti-CD80 FITC, anti-CD86 FITC or anti-PD-L1 Brilliant Violet 421 antibodies. HLA-A11 and HLA-A2 expression were measured by indirect staining with primary human anti-HLA-A11 and mouse anti-HLA-A2 antibodies, followed by secondary rabbit anti-human IgG FITC and goat anti-mouse IgG Alexa Fluor 647 antibodies, respectively. Dead cells were excluded using Zombie Aqua. Depicted is a representative staining of fluorescence minus FITC on bone marrow cells from mouse 1 treated with PBS. Viable single cells were gated and selected for tdTomato Red+ OCI-AML3.bm10 cells. Positive staining for the different markers within tdTomato Red+ OCI-AML3.bm10 cells was determined using FMO controls; (**C**) Bone marrow samples were analyzed for T-cell persistence. No T-cell persistence was observed in any of the treatment groups, with samples from mice receiving HLA-A11 dNPM1 T-cells (n=8, red squares), EBV T-cells (n=5, blue triangles) or HLA-A2 dNPM1 T-cells (n=4, green circles) and mice injected with PBS (n=2, black diamonds) showing similar background staining; (**D**) The frequency of OCI-AML3.bm10 cells in bone marrow samples was analyzed. The percentage of tdTomato Red+ OCI-AML3.bm10 cells was similar in bone marrow samples from mice treated with HLA-A11 dNPM1 T-cells (n=8, red squares) or EBV T-cells (n=5, blue triangles) and mice receiving PBS (n=2, black diamonds), but was lower in mice injected with HLA-A2 dNPM1 T-cells (n=4, green circles); (**E**) Surface expression of HLA class I, co-stimulation and adhesion molecules on bone marrow samples was assessed. AML cells from mice treated with HLA-A2 dNPM1 T-cells (n=4, green circles) showed slightly lower CD86 expression and higher HLA-A11 expression than AML cells from mice treated with HLA-A11 dNPM1 T-cells (n=8, red squares), EBV T-cells (n=5, blue triangles) or mice receiving PBS (n=2, black diamonds). ΔMFI was calculated by subtraction of the MFI of the FMO control for each sample. For HLA-A11 and HLA-A2, ΔMFI was calculated by subtraction of the MFI of the secondary antibody only. Horizontal lines depict mean ± SD.
